# The Good, the Bad and the New about Uric Acid in Cancer

**DOI:** 10.3390/cancers14194959

**Published:** 2022-10-10

**Authors:** Simone Allegrini, Mercedes Garcia-Gil, Rossana Pesi, Marcella Camici, Maria Grazia Tozzi

**Affiliations:** 1 Unità di Biochimica, Dipartimento di Biologia, Università di Pisa, Via San Zeno 51, 56127 Pisa, Italy; 2 Interdepartmental Research Center Nutrafood “Nutraceuticals and Food for Health”, Università di Pisa, 56126 Pisa, Italy; 3 CISUP, Centro per L’Integrazione della Strumentazione dell’Università di Pisa, 56127 Pisa, Italy; 4 Unità di Fisiologia Generale, Dipartimento di Biologia, Università di Pisa, Via San Zeno 31, 56127 Pisa, Italy

**Keywords:** uric acid, hyperuricemia, oxidative stress, cancer, fructose, xanthine oxidoreductase, AKT, AMPK, mTOR, uricase

## Abstract

**Simple Summary:**

The concentration of uric acid in blood is sex-, age- and diet-dependent and is maintained close to its maximal solubility, indicating that it plays some important role. Indeed, it has been demonstrated that, at physiological concentrations, uric acid is a powerful antioxidant and is a scavenger of singlet oxygen and radicals. At high intracellular concentration, uric acid has been demonstrated to act as a pro-oxidant molecule. Recently, uric acid has been reported to affect the properties of several proteins involved in metabolic regulation and signaling, and the relationship between uric acid and cancer has been extensively investigated. In this review, we present the most recent results on the positive and negative effects played by uric acid in cancer and some new findings and hypotheses about the implication of this metabolite in the pathogenesis of several diseases such as metabolic syndrome, diabetes, and inflammation, thus favoring the development of cancer.

**Abstract:**

Uric acid is the final product of purine catabolism in man and apes. The serum concentration of uric acid is sex-, age- and diet-dependent and is maintained close to its maximal solubility, indicating that it plays some important role. Indeed, it has been demonstrated that, at physiological concentrations, uric acid is a powerful antioxidant, while at high intracellular concentrations, it is a pro-oxidant molecule. In this review, we describe the possible causes of uric acid accumulation or depletion and some of the metabolic and regulatory pathways it may impact. Particular attention has been given to fructose, which, because of the complex correlation between carbohydrate and nucleotide metabolism, causes uric acid accumulation. We also present recent results on the positive and negative effects played by uric acid in cancer and some new findings and hypotheses about the implication of this metabolite in a variety of signaling pathways, which can play a role in the pathogenesis of diseases such as metabolic syndrome, diabetes, and inflammation, thus favoring the development of cancer. The loss of uricase in *Homo sapiens* and great apes, although exposing these species to the potentially adverse effects of uric acid, appears to be associated with evolutionary advantages.

## 1. Introduction

Uric acid (UA) is the final product of purine catabolism in humans and great apes [1,2]. Its concentration is maintained at high levels in blood through a mechanism of filtration and reabsorption, commonly between 178 and 360 μM (3 and 6.8 mg/dL). Higher levels are found in males and postmenopausal females [3]. UA, being a good electron donor, has a function as antioxidant in organisms that, like humans, are unable to synthetize ascorbate [4]. Soluble UA (SUA) levels higher than 360 μM are found in hyperuricemia, and 700 μM (about 13 mg/dl) or higher levels are usually associated with gout and acute kidney injury arising from the deposition of monosodium urate crystals in the renal tubules and interstitium [5]. A correlation between high levels of SUA and obesity, metabolic syndrome, diabetes, and inflammation has been described by different authors, casting new light on this metabolite, indicating that it can play a role as metabolic regulator and in the pathogenesis of several diseases including cancer [3]. In fact, some types of cancer appear to be strongly connected with inflammation, metabolic syndrome and obesity (breast, liver, colorectal, pancreatic, prostatic, renal, endometrial, ovarian, head, neck and esophageal cancer) [6,7,8,9,10,11,12,13,14,15,16,17]. However, it is presently unknown whether a common mechanism underlies these pathologies. In this regard, UA has been reported to act as a pro-oxidant molecule intracellularly and some of its adverse effects on liver have been associated with this property [18,19,20]. In this review, we summarize the possible causes of SUA accumulation or depletion and the metabolic and regulatory pathways on which it may impact. The levels of SUA are determined by several mechanisms: first, the production that depends on the rate of purine catabolism and recycling, with xanthine oxidoreductase (XOR) being the enzyme directly responsible for purine base conversion into UA. The efficiency of UA excretion and reabsorption depends on the involved transport systems. Furthermore, an increase in SUA level may arise from any mechanism leading to extensive purine catabolism, not only in the case of cell death but also as a consequence of the metabolism of a load of fructose. We also report recent results demonstrating that UA accumulation can start inflammation in different types of cells. Finally, we find particularly interesting current opinions on the mutations leading to the loss of uricase in *Homo sapiens* and great apes and the associated evolutionary advantages.

## 2. UA Homeostasis

**Figure 1:**
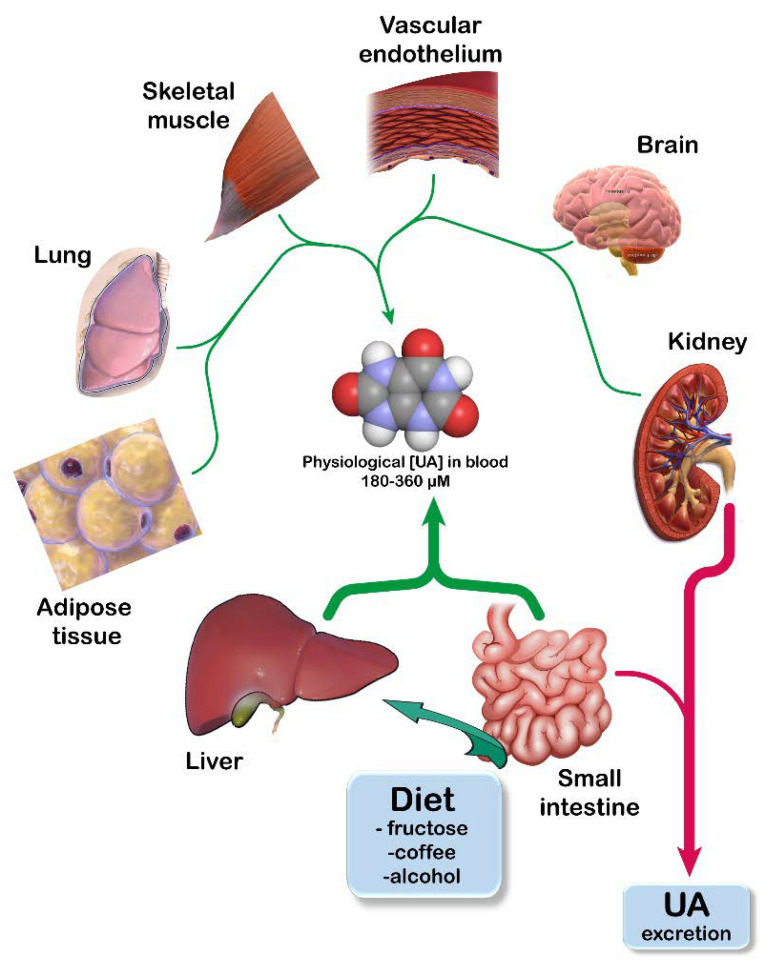
Uric acid (UA) homeostasis. UA is the final product of the catabolism of purine bases. UA homeostasis is maintained through a correct balance of its production (green arrows) and its excretion (red arrows). UA production occurs mainly in those tissues that express high levels of xanthine oxidoreductase (the enzyme catalyzing the last two steps of its biosynthesis). Kidneys account for about two thirds of the daily excretion of UA, while gut removes the remaining one third into the feces. Adapted from [27].

SUA homeostasis is maintained by an accurate balance between its income (either endogenous production or ingestion in diet) and its excretion. Any alteration of this system can lead to either hypouricemia or hyperuricemia. It is well known that the assumption of purine rich foods (such meat, poultry, some fishes, and vegetables), fructose, coffee, and alcohol increases SUA [21]. De Oliveira et al. [22] estimate that a rich purine diet could account for about 1-2 mg/dL SUA. Liver is the main organ in which adenylic and guanylic purine nucleotides, nucleosides, and bases (both of endogenous origin or coming from outside the liver, mainly released by degradation of dead cells) are catabolized to generate UA (Figure 1). Additionally, small intestine, adipose tissue, kidney, lung, muscle, brain, and vascular endothelium produce UA [21,23,24,25,26].

The enzyme responsible for the endogenous production of UA is XOR, which catalyzes the last two irreversible steps of purine catabolism in uricotelic animals, the oxidation of hypoxanthine to xanthine and of xanthine to UA. XOR is a homodimeric metalloflavoprotein with a molecular mass of approximately 300 kDa in which each subunit is composed of three domains: the 20 kDa N-terminal domain, with two identical iron–sulfur clusters, the 40 kDa intermediate domain with a FAD cofactor and the 85 kDa C-terminal domain with a molybdopterin cofactor containing a molybdenum atom. The C-terminal domain expresses nitrite reductase, xanthine dehydrogenase (XDH) and xanthine oxidase (XO) activities, while NADH oxidase activity resides in the FAD domain [28,29]. Therefore, the products of XOR activities are nitric oxide from nitrite reductase, UA and NADH from XDH, UA and reactive oxygen species (ROS) from XO, ROS from NADH oxidase. In turn, XDH can also generate ROS because the produced NADH is substrate of NADH oxidase activity of the FAD domain [29]. In humans, the highest XOR mRNA levels were found in liver and small intestine, the organs expressing the highest enzyme activity [30,31,32], while in mice, lung and adipose tissue have also been reported to express high XOR activity [26]. The physiological role and tissue distribution of XOR are extensively reviewed in Battelli et al. [33] and Bortolotti et al. [29]. Mammalian XOR is constitutively expressed as NAD+-dependent dehydrogenase and can be post-transcriptionally transformed in oxidase. This conversion may occur in a reversible way, through the oxidation of two cysteine residues [34,35] and irreversibly by limited proteolysis of the fragment containing such cysteine residues [36,37]. The mechanism of transition from XDH to XO has been detailed by Nishino et al. [38], who suggested the sophisticated mechanism of conversion being indicative that “the conversion is not a simple artefact, but rather has a function in mammalian organisms”. The involvement of XOR in cancer is recognized, and its expression and activity appear variable in tumors, where the enzyme has been shown to play either suppressive or oncogenic roles [39,40]. As recently reviewed by Chen et al. [40], the inhibition or upregulation of XOR may play a beneficial role in cancer therapy depending on the type of tumors.

The complex mechanisms of UA excretion have been thoroughly studied, and a plethora of transporters have been identified. For extensive and detailed coverage, the reader is referred to the numerous reviews on this subject [24,41,42,43,44]. UA is excreted mainly from the kidneys (about 70%) and, to a lesser extent, from the intestine (about 30%) [41,43,44,45]. SUA is freely filtered in the renal glomeruli from blood. Once in the lumen, up to 95% UA is reabsorbed; then, about 50% is excreted again, and finally, this is reabsorbed. After the second reabsorption process, a final fraction of about 5-15% of original UA is excreted in the urine. In the kidney, many transporters have been demonstrated to play a direct role in the excretion, including sodium-dependent phosphate cotransporters type 1 and 4 (NPT1, 4), organic anion transporters (OAT) 1-3, ATP-binding cassette transporter type G2 (ABCG2) [43,44] and type C4, also named multidrug resistance protein 4 (MRP4) [24,43]. Urate transporter type 1, OAT type 4 and 10 and glucose transporter type 9 (GLUT9) are reported to be involved in reabsorption [43,44]. Others are functionally coupled with UA transporters such as sodium-coupled monocarboxylate transporter 1 and sodium-dependent dicarboxylate transporter [43,44]. Although UA excretion from the gut accounts for only 30% of the total, its contribution becomes more relevant in the case of renal failure. The process of UA excretion in the intestine is much less characterized when compared to that of the kidney. Intestinal epithelial cells express several different kinds of transporters [44,46] that may be directly involved or functionally coupled with UA excretion. Those that are better characterized are ABCG2 and GLUT9. UA may enter the cell through GLUT9, which is located on the basolateral side of the epithelial cells and is then secreted into the intestinal lumen using ABCG2. Many other transporters have been identified in the intestinal epithelial cells, including multidrug resistance proteins (MRP2, MRP4, monocarboxylate transporter 9, NPT4, NPT5 and OAT10), but their role in the excretion process still needs to be clarified [44,46].

A relationship between defects in the structure and function of several urate transporters and pathologies has been described, particularly regarding hypertension and vascular diseases [24], and also chronic kidney disease [47]. A correlation between ABCG2 expression in tumor and an unfavorable prognosis has been reported. Indeed, substrates of this transporter, besides UA, are several anticancer agents [48]. Therefore, both impaired expression levels and variants of ABCG2, exhibiting defective transport functions, exert a significant effect on drug and UA metabolism, thus affecting the response to chemotherapeutic treatment (for a complete review of this issue, we direct the reader to the recent and complete review of Sarkadi et al. [48]).

## 3. UA and Fructose Metabolism in Cancer

**Figure 2:**
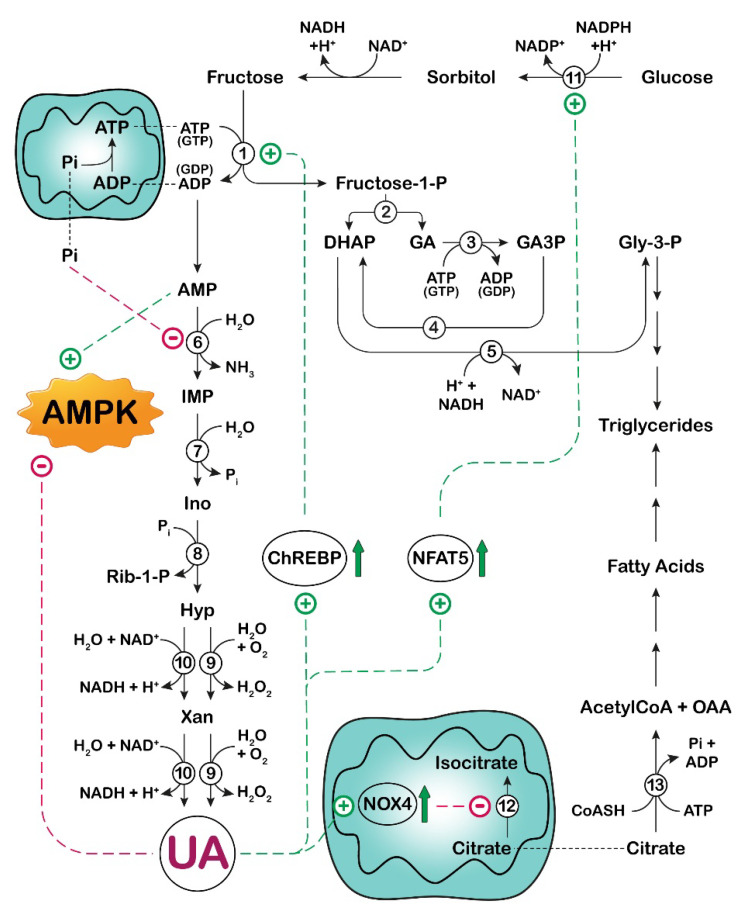
Effect of UA on fructose metabolism in liver. Phosphorylation of fructose in liver is catalyzed by fructokinase (enzyme 1), which is not subjected to feedback inhibition and phosphorylates fructose as rapidly as it can, leading to intracellular ATP and phosphate depletion. The formed fructose-1-phospahte is cleaved by aldolase B (enzyme 2) into dihydroxyacetone phosphate (DHAP) and glyceraldehyde (GA). GA is phosphorylated to glyceraldehyde-3-phosphate (GA3P) (triokinase, enzyme 3), which can enter the glycolytic pathway or can be converted by triose phosphate isomerase (enzyme 4) into DHAP. In turn, DHAP, by glycerol-3-phosphate dehydrogenase (enzyme 5), is reduced to glycerol-3-phosphate (Gly-3-P), which contributes to the synthesis of triglycerides. As a consequence of ATP catabolism, AMP is generated. The deamination of AMP, catalyzed by AMP deaminase (enzyme 6), is accelerated, and AMP is channeled towards IMP, which is hydrolyzed by cytosolic-5’-nucleotidase II (enzyme 7) to inosine (Ino). In turn, Ino is phosphorolytically cleaved into hypoxanthine (Hyp) and ribose-1-phosphate (Rib-1-P) by purine nucleoside phosphorylase (enzyme 8). Rib-1-P can be mutated to ribose-5-phosphate and then converted to 5-phosphoribosyl-1- pyrophosphate (see Figure 3), thus contributing to the increase in purine de novo synthesis. Hyp, through the action of xanthine oxidoreductase (oxidase, enzyme 9 or dehydrogenase, enzyme 10), is converted to UA. UA exerts an inhibitory effect on AMPK (see Figure 4) and an activatory effect (through stimulation of ChREBP) on fructokinase (enzyme 1). UA also activates aldose reductase (enzyme 11) (through stimulation of NFAT5), enabling endogenous fructose production from glucose. In addition, UA promotes the translocation of NADPH oxidase 4 (NOX4) from cytosol to mitochondria, which increases mitochondrial ROS generation with inactivation of aconitase (enzyme 12). Citrate is released by the mitochondrion and then converted back, by cytosolic ATP-citrate lyase (enzyme 13), to acetyl-CoA, necessary for the synthesis of fatty acids and triglycerides. ChREBP: carbohydrate response element binding protein; NFAT5: nuclear factor of activated T-cells 5; OAA: oxalacetic acid; Xan: xanthine; AMPK: AMP-activated protein kinase; 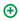: activation; 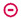: inhibition; 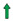: increase.

**Figure 3:**
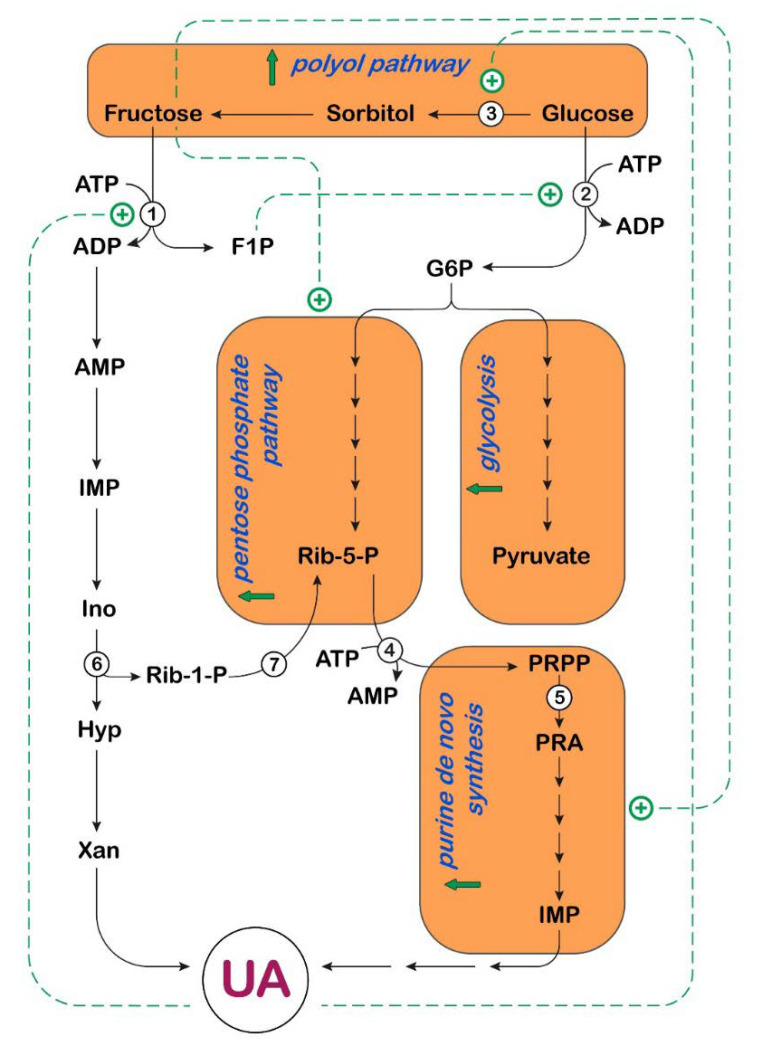
Effect of UA on carbohydrate and purine metabolism in liver. UA activates fructokinase (enzyme 1), increasing the formation of fructose-1-phosphate (F1P), which activates glucokinase (enzyme 2), by disrupting its interaction with glucokinase regulatory protein, contributing to the increase in the glycolytic flux. UA also activates aldose reductase (enzyme 3), enabling endogenous fructose for-mation through the polyol pathway. Fructose, through up-regulation of transketolase, favors the non-oxidative branch of the pentose phosphate pathway, for the synthesis of ribose-5-phosphate (Rib-5-P), essential for the formation of 5-phosphoribosyl-1-pyrophosphate (PRPP), a precursor of IMP, through the purine de novo synthesis pathway. The purine de novo pathway is also acceler-ated by depletion of adenine nucleotides, caused by a load of fructose (see Figure 2), which promotes a release in feedback inhibition of PRPP synthetase (enzyme 4) and amidophosphoribosyltransferase (enzyme 5). Rib-1-P generated during degradation of purine nucleotides by purine nucleoside phosphorylase (enzyme 6) is converted by phosphoribomutase (enzyme 7) to Rib-5-P, contributing to the acceleration of the purine de novo synthesis. G6P: glucose-6-phosphate; Ino: inosine; Hyp: hypoxanthine; Rib-1-P: ribose-1-phosphate; Xan: xanthine; PRA: phosphoribosylamine, 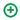: activation; 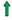: increase.

It has long been established that a load of fructose is accompanied by an increase in UA. In fact, as described below, fructose has been demonstrated to stimulate both the purine de novo synthesis and the breakdown of adenylic compounds. For this reason, in several published articles, UA is defined as a fructose metabolite. Indeed, none of its component atoms derive directly from fructose. To avoid any confusion, it would be better to point out that the increase in UA concentration, which follows a load of fructose, is a consequence of the complex array of correlation between carbohydrate and nucleotide metabolism (Figure 2 and Figure 3).

Raivio et al. [49] investigated, in their pioneering work, the association between fructose infusion, rate of purine de novo synthesis and urate excretion in humans. Based on the observation that the concentration of 5-phosphoribosyl-1-pyrophosphate (PRPP) was diminished in erythrocytes of individuals subjected to their investigation, they excluded that an increase in PRPP could account for the observed increased stimulation of purine synthesis. However, they were not able to measure the PRPP content in human liver [49]. Later on, a fructose-dependent increase in PRPP content was measured in the liver of mice [50] and rats [51]. In fact, as detailed below, a fructose load determines a decrease in concentration of adenine nucleotides, and therefore a release in feedback inhibition of amidophosphoribosyltransferase and PRPP synthetase, thus accounting for the accelerated purine de novo biosynthesis [50,51] (Figure 3).

In several organs, fructose is metabolized by a specific enzyme, called fructokinase (ketohexokinase, KHK), instead of being phosphorylated in the 6-position by hexokinases. KHK catalyzes conversion of fructose into fructose-1-phosphate, using ATP as phosphate donor. KHK is expressed as two splice variants, KHK-C and KHK-A [52]. The K_m_ for fructose of the C and A isoforms differ markedly, at approximately 0.5 and 8 mM, respectively. While KHK-A is widely expressed at low levels, KHK-C is selectively expressed in key metabolic tissues, such as liver, small intestine, and kidney [53]. For a better insight into the implications of the two isoforms in fructose metabolism, the reader is referred to recent and exhaustive reviews [54,55]. While hexokinases have negative feedback, which prevents excessive phosphorylation, KHK is not regulated and phosphorylates fructose as rapidly as it can, leading to intracellular ATP, GTP and phosphate depletion [56]. The phosphate of fructose-1-phosphate must necessarily come from ATP, therefore causing a decrease in the nucleotide concentration. However, the observed decrease in phosphate level is not immediately clear and can be explained by the rapid regeneration of ATP (through oxidative phosphorylation) at the expense of phosphate (Figure 2). The decreased concentration of GTP after a load of fructose has been explained by assuming that both triokinase (the enzyme that catalyzes the conversion of glyceraldehyde into glyceraldehyde-3-phosphate [56]) and KHK [57] can also utilize GTP as phosphate donor. As a consequence of ATP catabolism, AMP is generated, and its deamination, catalyzed by AMP deaminase (AMPD), is accelerated. In fact, the effect of the decreased concentration of the AMPD inhibitors, phosphate and GTP, prevails over the diminution of the activator, ATP [56]. Therefore, AMP is channeled towards IMP, which is hydrolyzed by cytosolic-5’-nucleotidase II (cN-II) to inosine (Ino). In turn, Ino is phosphorolytically cleaved into hypoxanthine (Hyp) and Rib-1-P by purine nucleoside phosphorylase (PNP) (Figure 2). Rib-1-P can be mutated to Rib-5-P and then converted to PRPP, thus contributing to the increase in purine de novo synthesis (Figure 3). Hyp, through the action of XOR, is converted to UA. Goncalves et al. [58] have identified KHK as the “key accelerator of tumor growth”. Indeed, they reported a rapid depletion of ATP in tumors exposed to both glucose and high-fructose corn syrup using a genetically engineered mouse model of intestinal tumorigenesis. They speculated that the reduction in ATP, caused by the rapid and uncontrolled phosphorylation of fructose by KHK, activated phosphofructokinase-1 and accelerated the flux of glucose through glycolysis, with a further reduction in phosphate (in the step of glyceraldehyde-3-phosphate dehydrogenase). This caused activation of AMPD, with subsequent degradation of the adenine nucleotide pool and UA formation. Fructose-1-phosphate in turn activated glucokinase by disrupting its interaction with glucokinase regulatory protein (GCKR) and exerting an opposite effect to that of fructose-6-phosphate [59]. Therefore, fructose-1-phosphate production contributed to the increase in the glycolytic flux (Figure 3). Goncalves et al. [58] explored the mechanism by which enhanced glycolysis increased tumor growth in their model and found that tumors reprogram their metabolism in favor of fatty acid synthesis, essential for cancer cell growth. In their work, the authors did not mention a direct link between fructose-derived UA and KHK activity [58]. In this regard, the expression of KHK has also been reported to increase significantly in hepatocytes of rats [60] and humans [61] exposed to fructose. Lanaspa et al. [62] reported that in fructose-fed rats, the up-regulation of liver KHK, observed both as protein expression and at the mRNA level, was prevented by allopurinol, a known inhibitor of XOR [63]. This indicates that UA can control, at a transcriptional level, the expression of the enzyme. The same authors directly added UA to human hepatocarcinoma cell line HepG2, a cell culture system that, in contrast with rat hepatocytes, lacks uricase. They observed a dose-dependent KHK up-regulation, independent of the presence of fructose [62]. With the aim to demonstrate the molecular mechanism underlying KHK activation by UA, Lanaspa et al. [62] explored the implication of the transcriptional factor carbohydrate response element binding protein (ChREBP), which is known to have KHK as one of its target genes [64] and is activated by fructose [65]. In this regard, they demonstrated that the fructose-dependent stimulation of the transcriptional activity of ChREBP in HepG2 cells was UA-dependent, since both the acetylation state of ChREBP and its nuclear translocation were reduced by allopurinol [62]. Interestingly, the acetylation state of ChREBP was significantly reduced not only in fructose-, but also in glucose-exposed human hepatocytes when allopurinol was present, probably because, as discussed later in this section, an overload of glucose caused endogenous fructose production [66]. ChREBP has also been demonstrated to up-regulate the fructose transporter Glut5 gene expression [67,68]. Therefore, the stimulation of ChREBP increases both fructose absorption and metabolism in the small intestine. In addition, Lanaspa et al. [20] reported that both fructose-derived and directly administered UA increased fat in human HepG2 cells. In fact, the authors demonstrated by confocal microscopy that, in HepG2 cells, UA promoted the translocation of NADPH oxidase 4 (NOX4) from cytosol to mitochondria, thus increasing mitochondrial ROS generation [20,69]. Mitochondrial oxidative stress has been demonstrated to inactivate aconitase [20,70]. Accumulated citrate was released by the mitochondrion and converted back, by cytosolic ATP-citrate lyase, to acetylCoA, a substrate for the synthesis of fatty acids. Additionally, ATP-citrate lyase activation by phosphorylation in HepG2 cells appeared to be dependent on the presence of fructose or UA [20]. However, the prevention of fructose-derived fatty acid synthesis by the addition of allopurinol indicated that UA was responsible for the process. Indeed, both fructose-dependent aconitase inactivation and ATP-citrate lyase activation were reverted by the presence of the XOR inhibitor [20]. 

Therefore, UA appears to be directly implicated in the activation of fructose metabolism, and in the channeling of fructose-derived metabolites into fatty acid synthesis. This is particularly relevant in the liver, where a dietary intake of fructose has been shown to be associated with non-alcoholic fatty liver disease [55]. 

UA also appears to influence the formation of endogenous fructose, through the activation of the polyol pathway (Figure 2 and Figure 3) [71]. Although reversible, the more physiologically relevant flux of this metabolic route appears to be from glucose to fructose, enabling endogenous fructose production. The rate-limiting enzyme in the pathway is aldose reductase (AR) [72], the involvement of which in the pathogenesis of diabetic complications is well-documented [73]. More recently, a direct link between AR expression and epithelial to mesenchymal transition in lung cancer patients has been reported [74], and a correlation between AR overexpression and breast, ovarian, cervical, and rectal cancer was discovered [75,76]. In human HepG2 cells, a UA-dependent up-regulation of AR was observed, and this activation appeared to be mediated by oxidative stress [71]. Indeed, the exposure of HepG2 cells to UA increased the nuclear expression of the transcription nuclear factor of activated T-cells 5 (NFAT5), resulting in the up-regulation of one of its targets, the AR gene [77] (Figure 2 and Figure 3). Indeed, despite being an important antioxidant extracellularly [4], UA can act as a pro-oxidant molecule intracellularly [18,19,20]. In this regard, Wang et al. [78] demonstrated that AR was up-regulated in liver specimens from patients with alcoholic hepatitis, with consequent elevation in fructose, sorbitol, and UA. By correlation analyses, they showed an association between AR expression (or fructose, sorbitol, and UA levels) and the hallmark ER-stress gene activating transcription factor 3 (ATF3) and CCAAT/enhancer-binding homologous protein (CHOP) [78]. Therefore, the UA-dependent up-regulation of AR shifts glucose fate from glycolysis to endogenous fructose production in hyperuricemic conditions and high glucose diet. Elevated UA potentiates the effect of high glucose in the activation of the polyol pathway with subsequent triglyceride accumulation in the liver. 

Overall, fructose has been reported as an alternative energy source, exploited for tumor growth. Its utilization appears preferential under low oxygen conditions [79] and accelerates glucose utilization [58,80]. UA and lactate appear to be its major by-products. Fructose has also been shown to be used for nucleic acid synthesis in cancer cells. Liu et al. [81] demonstrated, using human pancreatic cancer cells, that fructose was preferentially channeled (with respect to glucose) to the non-oxidative branch of the pentose phosphate pathway, for the synthesis of additional nucleic acids, through the up-regulation of transketolase. This is critical to support the high proliferation rates of cancer cells [82]. Even though a direct involvement of UA in the activation of the pentose phosphate pathway was not reported, a fructose-dependent increased production of UA was demonstrated in pancreatic cancer cells [81]. All the above-described effects converge to promote cell proliferation and cancer growth (Figure 3).

## 4. UA and AMPK in Cancer

Lipid accumulation in the liver (hepatic steatosis) might lead to steatohepatitis and, finally, to cirrhosis [83]. The persistent accumulation of lipids in the liver can cause inflammation and metabolic impairment and create a pro-metastatic niche [84]. Many studies have found an association between hepatic steatosis and liver metastasis of colorectal cancer [85,86,87], while others did not find this correlation [88].

**Figure 4:**
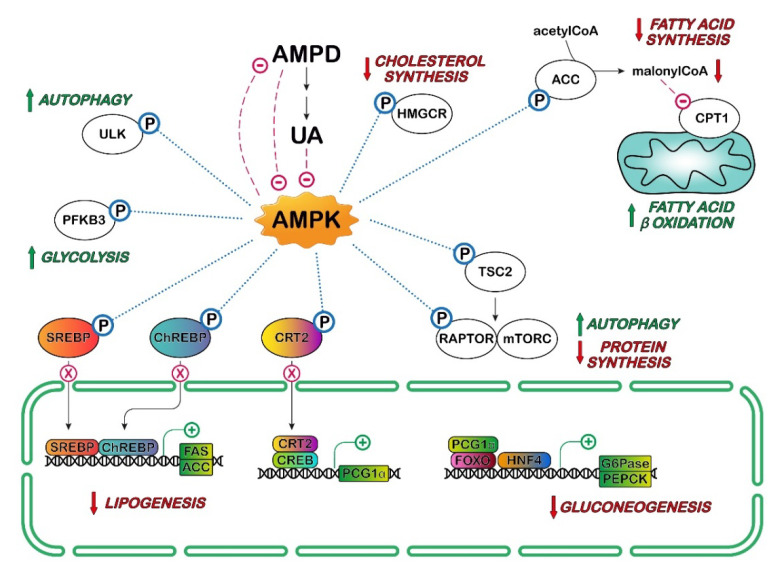
Effect of AMP-activated protein kinase (AMPK) on several metabolic pathways. Activation of AMPK represses anabolic processes by affecting the activities of several proteins which play crucial roles in protein, fatty acid, sterol, and cholesterol synthesis: target of rapamycin complex 1 (mTORC1), 3-hydroxy-3-methyl-glutaryl-coenzyme A reductase (HMGR), acetyl-CoA carboxylase (ACC), sterol regulatory element-binding protein (SREBP), carbohydrate response element binding protein (ChREBP). In addition, AMPK stimulates catabolic processes to produce ATP. The phosphorylation of CREB-regulated transcription coactivator 2 (CRT2) may allow for its sequestration into the cytoplasm and prevents its nuclear translocation, reducing the expression of peroxisome proliferator-activator receptor-gamma coactivator 1-alpha (PGC-1α). In association with the transcription factors hepatocyte nuclear factor 4 alpha (HNF4) and forkhead box protein O1 (FOXO), PGC-1 α stimulates the transcription of genes codifying glucose-6-phosphatase (G6Pase) and phosphoenolpyruvate carboxykinase (PEPCK) involved in gluconeogenesis. AMPK increases glucose flux along the glycolytic pathway by phosphorylation of 6-phosphofructo-2-kinase/fructose-2,6-bisphosphatase 3 (PFKFB3), which affects the phosphofructokinase-1 activity, a rate-limiting enzyme in glycolysis. The phosphorylation by AMPK and inhibition of ACC results in lower levels of malonyl-CoA, which is an allosteric inhibitor of carnitine palmitoyltransferase-1 (CPT1). This indirectly stimulates the transport of fatty acids into the mitochondria, increasing fatty oxidation, while inhibiting fatty acid synthesis. AMPK induces autophagy both directly by Unc-51-like autophagy activating kinase 1 (ULK1) phosphorylation, and indirectly by mTORC1 inactivation (see Figure 6 for further details). AMP deaminase (AMPD) and its metabolite UA inhibit AMPK, while AMPK inhibits AMPD (see text for the metabolic implications). CREB: cAMP response element-binding protein; FAS: fatty acid synthase; mTOR: mammalian target of rapamycin complex; RAPTOR: regulatory-associated protein of mTOR; TSC2: tuberous sclerosis complex; 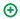: activation; 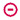: inhibition; 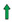: increase; 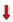: decrease. 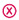 indicates lack of nuclear translocation and therefore loss of transcription of the target genes.

Hepatic steatosis is associated with a reduction in AMP-activated protein kinase (AMPK) activity. AMPK-dependent phosphorylation of transcription factors such as sterol regulatory element-binding protein (SREBP)-1c, SREBP-2 and ChREBP regulate lipogenesis in liver. The phosphorylation of these factors leads to the inhibition of the transcription of genes coding for fatty acid synthase, acetyl-CoA carboxylase (ACC1) and stearoyl-CoA desaturase. AMPK can phosphorylate ACC1 at Ser79, resulting in its inactivation and reduction in lipogenesis (Figure 4). In addition, AMPK stimulates fat oxidation both by inhibiting ACC1 activity (thus decreasing the level of malonyl-CoA) and by increasing the transcription of peroxisome proliferator-activated receptor (PPAR)α and its downstream target genes [89,90,91].

It has been demonstrated that AMPK is inhibited when AMPD is activated in the HepG2 cell line, and that silencing of AMPK results in an increase in AMPD activity [92]. Lanaspa et al. [92] demonstrated that UA, which can be generated from AMP through the action of AMPD, cN-II, PNP and XOR (Figure 2), is an inhibitor of AMPK in this cell line. HepG2 cells accumulated fat upon exposure to fructose, and this effect appeared to be due to a predominant action of AMPD over AMPK. This was demonstrated in a series of elegant experiments by adding fructose to the culture medium of HepG2 cells in which AMPK or AMPD were silenced and by comparing the effect of fructose in non-silenced cells. Fructose exposure resulted in the higher activation of AMPK and in an increase in fatty acid oxidation when AMPD was silenced, and therefore, there was no UA-dependent AMPK inhibition. There was an increase in AMPD-dependent fat accumulation and a decrease in fatty acid oxidation when AMPK was silenced. In vivo, sucrose-fed rats were found to develop fatty liver, and AMPK activator metformin was able not only to increase AMPK activity and fatty acid oxidation, but also to inhibit AMPD activity and to reduce intrahepatic triglyceride accumulation. More recently, it was found that an acute fructose load in fructose-fed rats increased not only plasma and hepatic UA, but also hepatic and plasma triglycerides and hepatic oxidative stress. Fructose feeding was also associated with decreased AMPK, while KHK and XOR were overexpressed, and increased activity or expression of several enzymes involved in liver lipogenesis and activation of endothelial nitric oxide synthase (eNOS) was observed. Allopurinol treatment prevented hepatic and systemic alterations [93]. Garcia-Arroyo et al. [93] proposed that XOR inhibitors might delay the progression of non-alcoholic fatty liver disease, but this therapeutic effect needs to be proven in more appropriate long-term fed models.

Cicerchi et al. [94] have also demonstrated that the generation of Ino and UA induced by AMPD could contribute to the increase in glucose production in HepG2 cells. The inhibitory action of AMPD-derived UA on AMPK could prevent the phosphorylation of CREB-regulated transcription coactivator 2 (CRT2) and therefore allow for its translocation to the nucleus. This leads to the transcription of the rate-limiting enzymes of the gluconeogenesis, phosphoenolpyruvate kinase (PEPCK) and glucose-6-phosphatase, justifying the increase in glucose generation found in diabetes [94] (Figure 4). Cicerchi et al. [94] proposed that AMPD could be a druggable target for diabetes, in addition to the stimulation of AMPK. Many studies suggest that diabetes mellitus is a risk factor for several types of cancers and that it is associated with higher cancer mortality [95].

Wang et al. [96] suggested that preconditioning with UA could be protective for cardiomyocytes treated with the antineoplastic drug doxorubicin both in vivo and in vitro. Experiments using 5-aminoimidazole-4-carboxamide ribonucleotide (AICAR) and compound C (activator and inhibitor of AMPK, respectively) suggested that the mechanism of protection involved activation of AMPK and Src homology 2 domain-containing protein tyrosine phosphatase (SHP2), which resulted in reduction in doxororubicin-induced phosphorylation of c-Jun N-terminal kinases (JNK) and of the gap-junction protein connexin 43 [96], thus alleviating alterations in cardiomyocyte functionality.

The molecular mechanisms underlying activation and/or inhibition of AMPK by UA are still unknown, but the experiments described above indicate that the modification of AMPK activity, including the use of XOR inhibitors, could be useful either to delay hepatic steatosis that could ultimately lead to cancer or to alleviate chemotherapeutic-induced toxicity.

## 5. UA, Inflammation and mTOR/AKT

**Figure 5:**
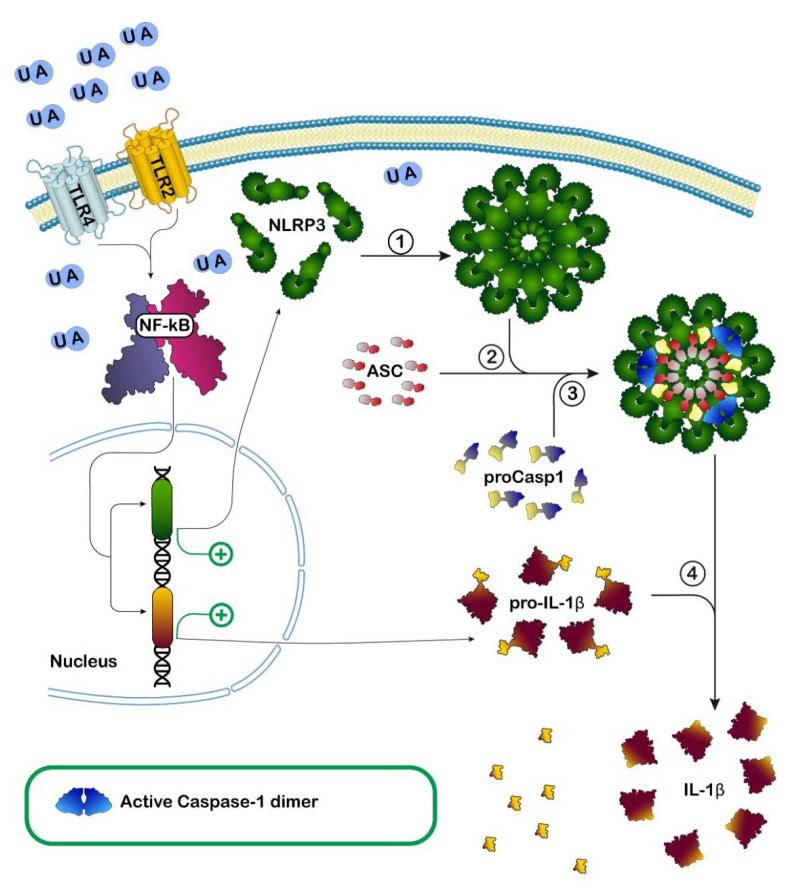
Effect of uric acid (UA) on inflammation. Proposed model in which Toll-like receptors 2 and 4 (TLR2, TLR4) signaling mediates induction of inflammation by both intracellular and extracellular UA crystals. In this model, based on the results of several studies, UA engages TLR2 and TLR4 receptors causing activation of the transcription factor nuclear factor kappa-light-chain-enhancer of activated B cells (NF-kB), which, in turn, promotes the increase in both Nod-like receptor family pyrin domain containing 3 (NLRP3) and pro-interleukin-1-beta (pro-IL-1β) synthesis. Activation, caused by different signals including UA, promotes NLRP3 oligomerization (1), the growing oligomer recruits and binds apoptosis-associated speck-like protein containing a caspase recruitment domain (ASC) (2), which subsequently recruits and binds pro-caspase-1 (proCasp1) (3). The activation and formation of NLRP3 inflammasome leads to auto-cleavage and formation of the active caspase-1 dimer which then proteolytically cleaves pro-IL-1β to its bioactive form IL-1β (4) prior to its release, thus starting the inflammatory response.

UA has been described as an endogenous danger signal released by necrotic cells able to activate adaptive immune responses [97]. Indeed, it has been demonstrated that UA crystals may interact with Toll-like receptors (TLR), membrane-bound receptors involved in innate immunity, causing inflammation. In particular, TLR-2, myeloid differentiation factor 88 (MyD88), and TLR-4 are involved in inflammatory responses to monosodium urate crystals by macrophages in vitro [98]. UA crystals possibly interact directly with the receptors, leading to the activation of transduction signaling, culminating with NF-kB activation. NF-kB is a transcription factor promoting the transcription of several proteins including pro-interleukin-1 (pro-IL-1) involved in inflammation [99]. Furthermore, it was shown that monosodium urate crystals and calcium pyrophosphate dehydrate crystals are potent activators of caspase-1 via the NOD-, LRR- and pyrin domain-containing protein 3 (NLRP3) intracellular inflammasome [100] (Figure 5).

The activation of intracellular inflammasome is usually triggered by bacterial proteins such as flagellin, but also by other endogenous signals. Not only UA crystals, but also other large particulate elements (e.g., asbestos, silica, and alum), can induce inflammation through NLRP3 inflammasome [101], suggesting that this branch of the innate immune system might have evolved to detect pathogenic particulates. NLRP3 inflammasome is a complex of proteins detecting, as mentioned before, bacterial components, but also other signals, leading to caspase-1 activation and IL-1 maturation, by the cleavage of pro-IL-1, thus recruiting the cellular immunity system. How UA crystals activate this inflammasome is not clear and is a matter of discussion. A current opinion is that the activation is actually mediated by ROS induced somehow by the presence of intracellular crystals [102].

In summary, the pro-inflammatory action of UA is mediated by both intracellular and extracellular receptors involved in innate immunity.

The activation of the mechanism of innate immunity by UA crystals is involved in the pathogenesis of gout and explains most of the symptoms present in patients [103].

The formation of UA crystals is commonly caused by an increase in circulating UA, usually due to a defect in its excretion and/or reabsorption [103] and also by an incorrect diet [104]. In fact, it was demonstrated that dietary patterns rich in animal or sweet foods were positively associated with a higher risk for hyperuricemia, whereas the vegetable pattern was negatively associated [105]. Hyperuricemia has been associated with a variety of pathological conditions such as metabolic syndrome and coronary artery disease, all associated, from low to high degree, with sterile inflammation [106,107]. Therefore, it was postulated that not only urate crystals, but also high concentrations of UA, could be responsible for activation of innate immunity system. In the last few years, UA has been shown to activate the TLR4-NLRP3 inflammasome and phosphatidylinositol-3-kinase (PI3K)/AKT signaling pathway, which is also involved in the regulation of UA gut excretion mediated by ABC transporters [108]. The ability to activate the inflammasome complexes constitutes the link between UA and acute inflammation associated with diseases such as gout and chronic inflammation [109]. Therefore, there is also a possible causal or con-causal link between hyperuricemia and other pathologies that have been associated with inflammation, such as cancer. Indeed, there is a vast literature demonstrating a positive correlation between UA concentration and incidence of cancer and/or outcome after therapy, although the correlation appears to be dependent on the type of cancer [3,110,111,112,113]. Most of the studies concern clinical observations, not providing any cue on the underlying molecular mechanisms. Correlation between high UA and incidence appears to be more significant in females than in males for pancreatic cancer, while the opposite was found for gall bladder cancers [114]. In addition to gastro-intestinal cancers, high SUA levels have also been associated with the incidence of urological cancers [115]. Indeed, gout patients have a higher risk of prostate, esophageal, stomach, colon, liver, pancreatic, lung, ovarian, renal, and bladder cancers, but not of breast or brain cancers [116,117]. Interestingly, both hypo- and hyper-uricemia are associated with high liver cancer risk (U-shaped association) [114]. Low UA has been found in the blood of patients with laryngeal squamous cell cancer [118]. In these patients, higher UA concentrations correlated with longer overall survival.

**Figure 6:**
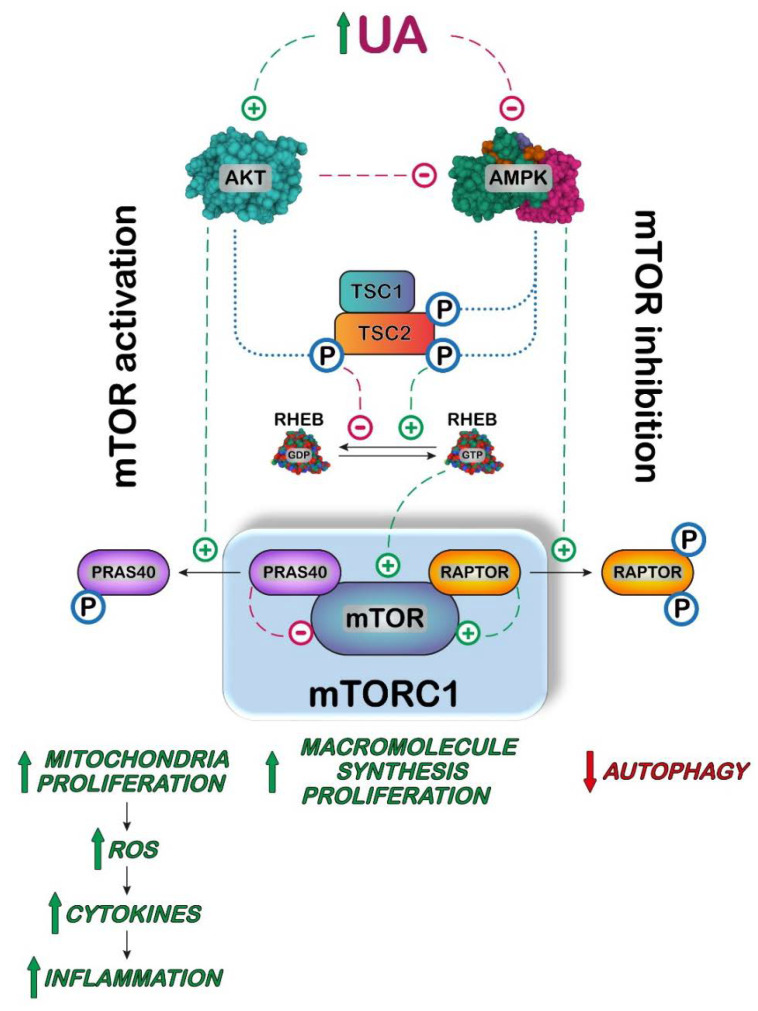
Proposed model in which UA activates mammalian target of rapamycin complex 1 (mTORC1). In several cell models, high UA concentration causes an increase in AKT activity and/or a decrease in AMPK activity causing a potentiation of the regulatory pathway leading to mTORC1 activation. Activated AKT phosphorylates tuberous sclerosis complex 2 (TSC2) leading to the release of the dimer tuberous sclerosis complex 1/2 (TSC1/TSC2) from the mTORC1 complex. Since the dimer is an activator of the GTPase activity of RAS homologous enriched in brain (RHEB), this G-protein binds GTP for long time, thereby activating mTORC1. AKT also directly activates mTORC1 by phosphorylating the mTOR inhibitor proline-rich AKT substrate of 40 kDa (PRAS40), which is then released from the complex. On the contrary, phosphorylation of TSC2 by AMPK increases the concentration of the dimer TSC1/TSC2, thus favoring the GTPase activity of RHEB and therefore inactivating mTORC1. The inhibitory activity of AMPK is also exerted by phosphorylation and consequent release of the mTOR activator regulatory associated protein of mTOR (RAPTOR). Therefore, activation of AKT and inhibition of AMPK cause an increase in mTORC1 activity leading to an increase in the rate of synthesis of proteins, lipids, and nucleotides, while autophagy is inhibited. mTORC1 activation also promotes mitochondria proliferation, producing an increase in intracellular ROS, responsible for an increase in cytokines and chemokines production, leading to inflammation. 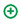: activation; 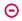: inhibition; 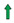: increase; 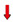: decrease.

The activation of the inflammatory mechanism and neoplastic transformation are accompanied in many cases by activation of the mTOR pathway. In fact, the protein kinase mTOR forms a complex with other proteins, some of which are involved in its functional connection with the amount and nature of ingested food [119,120]. With this in mind, it is particularly interesting to ascertain if UA is involved in the activation of the AKT/AMPK/mTOR pathway. It was demonstrated that high concentrations of UA are priming human monocytes, basically causing an increase in IL-1, favoring a proinflammatory phenotype in these cells [121]. Furthermore, AKT was phosphorylated to its active form, in primed monocytes incubated with high UA concentrations. This activation is followed by the phosphorylation of proline-rich AKT substrate 40 (PRAS40), one of the components of mTOR complex 1 (mTORC1), which can end up with mTORC1 activation [121] (Figure 6).

Indeed, UA priming causes a decrease in autophagic mechanism in HeLa cells [121]. Autophagy is a conserved housekeeping lysosomal process, which has the role of degrading long-lived intracellular cargos, but it is also closely linked to inflammation [122]. mTORC1 is the major inhibitor of autophagy, and its activity is stimulated by AKT [123]. On the other hand, activated AMPK phosphorylates regulatory-associated protein of mTOR (RAPTOR), causing dissociation of this mTOR activator with autophagy activation [124]. As UA is both an activator of AKT and an inhibitor of AMPK, high levels of this metabolite favor inflammation and inhibit autophagy (Figure 6). Conversely, UA has been indicated as an inhibitor of AKT phosphorylation in different kinds of cells such as cardiomyocytes and liver cells through a ROS-dependent pathway [125,126]. These contrasting results confirm the dual role played by UA in oxidative stress [127]. The relationship between UA and the function of the AKT/AMPK/mTOR pathway and/or inflammation has been investigated in vivo in transgenic mice silenced for uricase by different authors [128,129]. In this experimental model, high SUA concentrations promote atherosclerosis in the presence of cholesterol crystals, inducing the activation of NLRP3 inflammasome. Kimura et al. [128] demonstrated that UA inhibited AMPK phosphorylation with a consequent activation of mTOR, which, in turn, promoted mitochondrial activity producing ROS. Mitochondrial ROS led to an increase in HIF-1α activation, followed by increase in pro–IL-1β expression, IL-1β production, and NLRP3 inflammasome activation. The inhibition of mTOR or activation of AMPK reverted all the effects exerted by UA [128].

Overall, most of the reports indicate that UA activates the AKT/mTOR pathway, leading to an increase in all the anabolic pathways including lipid, protein and nucleotide synthesis and inhibiting autophagy, thus favoring cell proliferation. Furthermore, it increases inflammatory mechanisms, lipid circulation and storage, generating a metabolic disarrangement that, if constantly present, strongly increases the risk of all site cancer, as demonstrated by several reports [3,130].

## 6. UA and the Lack of Uricase Hypothesis

Excess of UA has always been considered a danger signal, being correlated to an incorrect diet, defective transport systems, or a surplus of ATP hydrolysis not compensated by oxidative phosphorylation [3], and thus is a negative signal. On the other hand, UA, as a good antioxidant [4,131], is maintained in blood at high concentrations and has been indicated to be responsible for the high life span and large brain present in humans and great apes [132], which is definitely a positive signal. The question is why, among mammals, do only humans and great apes accumulate UA if it can be so dangerous?

UA is the final product of the catabolism of purine nucleotides in humans and apes, whereas all other mammals further process this metabolite to produce allantoin [132]. This is due to two distinct loss-of-function mutations, which occurred 15.4–9.8 million years ago, in the gene coding for uricase, the enzyme that catalyzes the conversion of UA in 5-hydroxyisourate [1,2]. It has been hypothesized that uricase inactivation and thereby the increase in SUA could have caused an evolutionary advantage in lineages, such as ancient apes, that had recently lost their ability to synthesize ascorbate [133] and faced a prolonged starvation period during the global cooling of the middle Miocene [127]. Our ancestor diet was mainly composed of fruit and vegetables, poor in purines and rich in sucrose, a disaccharide constituted by glucose and fructose. These two sugars have different metabolic destiny: while glucose is utilized to give metabolic energy or is accumulated as a polymer (glycogen) in the liver and muscle and as fatty acids under the control of hormones, fructose enters glycolysis but is mainly converted into lipids (refer to the specific section of this review for a detailed description of the mechanisms). The different destinies of the two sugars are probably determined by their structure [134]. Glucose, being the more stable six-carbon sugar, has become the major energy supply and storage material precursor in living organisms, and its metabolism is maintained under control through many regulatory mechanisms. The metabolism of fructose, on the other hand, is not finely regulated; an overload of this carbohydrate causes an increase in UA, derived from uncontrolled ATP hydrolysis. Lines of evidence suggest that UA could increase the capability of both the absorption and catabolism of fructose in lineages deprived of uricase, resulting in a more efficient storage of nutrients in fat, a clear survival advantage for apes in the middle Miocene [127,135]. Nowadays, in countries where food is largely accessible, and medical assistance and drug availability increase the life span, we observe a chronic exposure to excess of both glucose and fructose, with a concomitant chronic increase in circulating UA. This promotes the synthesis, circulation, and storage of lipids, increasing the risk for diabetes, metabolic syndrome, chronic inflammation, and cancer. In this regard, Fini et al. [136] injected breast cancer cells in mice whose uricase was inactivated by either knocking out the gene or by an uricase inhibitor, and found that these treatments resulted in a remarkable increase in tumor growth and metastases. Conversely, transgenic mice overexpressing uricase showed a reduction in both SUA and tumor growth.

## 7. Conclusions

The relationship between high UA and cancer has been suggested in many papers, although a causal connection is difficult to demonstrate, due to several confounding factors such as diet, alcohol consumption, and underlying co-morbidity disorders. Furthermore, hyperuricemia, rather than being an independent risk factor for cancer, could be the result of cancer-related cell death [3]. Nevertheless, increasing lines of evidence indicate that the maintenance of urate homeostasis is very important for the wellness of the whole organism. In fact, both hypo- and hyper-uricemia have been correlated to an increase in cancer risk. Indeed, many cohort studies show a U-shaped association between SUA levels and increase in all-cause mortality, including cancer [137,138,139]. The inflection point for the curve was found at a SUA level of 6 mg/dL in males, and 4 mg/dL in females, corresponding to the physiological SUA levels [114]. The risk of contracting severe diseases in the presence of high SUA is explained by the regulatory function exerted by UA described above, while the danger related to hypouricemia might be due to the lack of the protective antioxidant effect exerted by physiological UA concentrations [140]. 

In conclusion, the maintenance of a correct balance of SUA appears to be necessary to decrease the risk of severe diseases, including cancer. Many factors contribute to SUA homeostasis: transport systems, XOR activity, and eating behavior. In modern society, the excessive intake of fructose, present in industrial food and beverages, appears to represent a real danger for public health.
